# Crouching Tiger, Hidden Protein: Searching for Insecticidal Toxins in Venom of the Red Tiger Assassin Bug (*Havinthus rufovarius*)

**DOI:** 10.3390/toxins13010003

**Published:** 2020-12-22

**Authors:** Laura C. Wait, Andrew A. Walker, Glenn F. King

**Affiliations:** Institute for Molecular Bioscience, The University of Queensland, 306 Carmody Rd, St Lucia, QLD 4072, Australia; l.wait@uq.net.au

**Keywords:** venom, toxin, assassin bug, Reduviidae, insecticidal, CUB domain, cystatin

## Abstract

Assassin bugs are venomous insects that prey on other arthropods. Their venom has lethal, paralytic, and liquifying effects when injected into prey, but the toxins responsible for these effects are unknown. To identify bioactive assassin bug toxins, venom was harvested from the red tiger assassin bug (*Havinthus rufovarius*), an Australian species whose venom has not previously been characterised. The venom was fractionated using reversed-phase high-performance liquid chromatography, and four fractions were found to cause paralysis and death when injected into sheep blowflies (*Lucilia cuprina*). The amino acid sequences of the major proteins in two of these fractions were elucidated by comparing liquid chromatography/tandem mass spectrometry data with a translated venom-gland transcriptome. The most abundant components were identified as a solitary 12.8 kDa CUB (complement C1r/C1s, Uegf, Bmp1) domain protein and a 9.5 kDa cystatin. CUB domains are present in multidomain proteins with diverse functions, including insect proteases. Although solitary CUB domain proteins have been reported to exist in other heteropteran venoms, such as that of the bee killer assassin bug *Pristhesancus plagipennis*, their function is unknown, and they have not previously been reported as lethal or paralysis-inducing. Cystatins occur in the venoms of spiders and snakes, but again with an unknown function. Reduction and alkylation experiments revealed that the *H. rufovarius* venom cystatin featured five cysteine residues, one of which featured a free sulfhydryl group. These data suggest that solitary CUB domain proteins and/or cystatins may contribute to the insecticidal activity of assassin bug venom.

## 1. Introduction

Assassin bugs belong to the hemipteran family Reduviidae. Most assassin bugs use their proboscis to pierce prey, inject venom, and feed from the envenomated prey. An exception is the kissing bugs, which are blood-feeding ectoparasites, whose venom disrupts host haemostatic defences [1]. Unlike kissing bugs, entomophagous assassin bugs are descended from ancestors that have retained predation as their principle trophic strategy for >200 million years [2]. Over this time, evolution has honed the insecticidal capability of assassin bug venom to contain potent and quick-acting insecticidal compounds. In 1961, Edwards [3] showed that the first instar larva of the assassin bug *Rhynocoris carmelita* could paralyse an insect greater than 400 times its weight (a first instar Mediterranean flour moth, *Ephestia kuehniella*) in under 10 s. The red spot assassin bug *Platymeris rhadamanthus* can paralyse the American cockroach *Periplaneta americana* sufficiently to stop its struggling within 3–5 s, and completely paralyse it within 15 s. Injection of 0.1 µl of venom from the bee-killer assassin bug *Pristhesancus plagipennis* obtained by electrostimulation, which is secreted by the large posterior main venom gland, paralyses third-instar crickets within seconds [4]. However, compared to snake, cone snail, and arachnid venoms, the toxins underlying these effects in assassin bug venoms are poorly characterised.

The ability of arachnid venoms to induce prey paralysis is due to their ability to modulate the function of ion channels on the surface of excitable cells [5,6]. The major bioactive components of most spider venom are disulfide-rich peptide neurotoxins that bind to and modulate presynaptic or postsynaptic neuronal ion channels. Assassin bug venoms also contain disulfide-rich peptide neurotoxins, such Ptu1 from the peiratine assassin bug *Peirates turpis*, which inhibits the voltage-gated calcium channel Ca_V_.2.2 [7]. Ptu1 has an inhibitor cystine knot (ICK) fold [8], which has been independently recruited into venoms produced by phylogenetically diverse animal groups, including arachnids and cone snails [9]. Previously, we reported that insecticidal venom of the assassin bug *P. plagipennis* contained multiple peptides with primary structures suggesting that they adopt an ICK fold [10]. However, in that study, we also reported that such peptides made up a small proportion (1–3%) of *P. plagipennis* venom, whereas many of the most abundant venom components were of unknown function. Moreover, injection of Ptu family peptides from assassin bug venom into prey species has been performed, but did not result in paralysis [7,11]. This suggests that other components underlie the paralytic effects of assassin bug venom.

**Figure 1:**
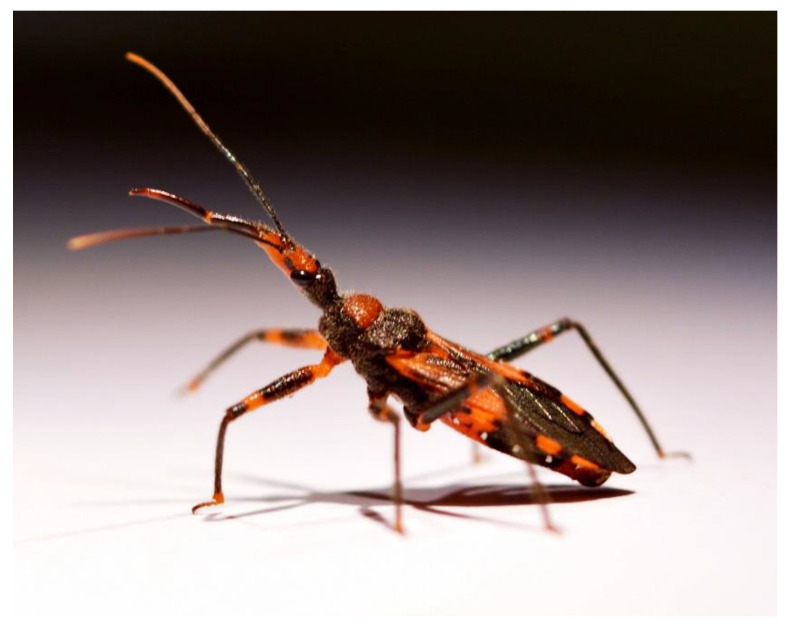
Red tiger assassin bug *H. rufovarius*. This harpactorine species is common under the bark of *Eucalyptus* sp. trees and is widespread in Australia. The individual shown is displaying a threat posture with proboscis extended in response to provocation with tweezers. Photo © Jiayi Jin.

In this study, we aim to better understand the venom components responsible for the paralysing and lethal effects of reduviid venoms by isolating insecticidal compounds from the venom of the red tiger assassin bug *Havinthus rufovarius* (Figure 1). We show that two of the most potent paralytic venom fractions contain a solitary CUB domain protein and a cystatin, neither of which has previously been reported to possess insecticidal activity.

## 2. Results

### 2.1. Electrostimulation and Harassment Yield Similar Venom

**Figure 2:**
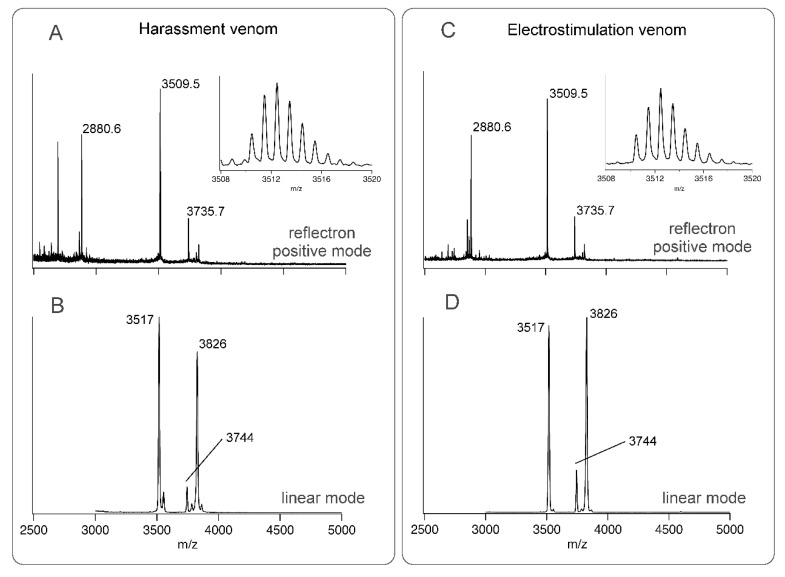
Matrix-assisted laser desorption/ionisation time-of-flight (MALDI-TOF) mass spectra of: (**A**) Venom obtained by harassment, taken in reflectron positive mode; (**B**) Venom obtained by harassment, taken in linear mode; (**C**) Venom obtained by electrostimulation, taken in reflectron positive mode; (**D**) Venom obtained by electrostimulation, taken in linear mode.

Since it has previously been reported that electrostimulation and harassment of assassin bugs can yield different venoms from different gland lumens [4,12,13], we investigated if this was also true for *H. rufovarius* (Figure 1). Although venom could be obtained by either stimulus, venom obtained by electrostimulation and harassment of *H. rufovarius* yielded similar mass profiles using matrix-assisted laser desorption/ionisation time-of-flight mass spectrometry (MALDI-TOF-MS) using a 5800 MALDI-TOF instrument (SCIEX, Framingham, MA, USA) (Figure 2). 

This may be due to the complexity of the nerve-muscle systems involved in regulating venom injection [4] or species-specific differences among assassin bugs [12,13,14]. The spectra obtained revealed venom components with monoisotopic masses of 3735.7, 3826, 3509.5, and 2880.6 Da that may represent venom peptides. Since both collection techniques yielded similar material, venom was collected by electrostimulation for the remainder of the study due to greater ease of attainment.

### 2.2. Isolation of Insecticidal Fractions

To investigate the physiological role of individual venom components, we deconvoluted crude *H. rufovarius* venom using reversed-phase high-performance liquid chromatography (RP-HPLC) to obtain 104 fractions (Figure 2). The RP-HPLC chromatogram had similarities to those we reported for the venom of the assassin bug *P. plagipennis* [10], in that >40% acetonitrile was required to elute the major venom components. In contrast, a typical fractionation of spider venom performed under comparable conditions with the same buffers typically yielded multiple peaks that eluted in <40% acetonitrile, and these overwhelmingly corresponded to peptides <10 kDa [15]. If these abundant components of *H. rufovarius* venom are proteins >10 kDa, as in *P. plagipennis* venom, this result will highlight the difference in the mass distribution of venom components between different classes of venomous arthropods.

**Figure 3:**
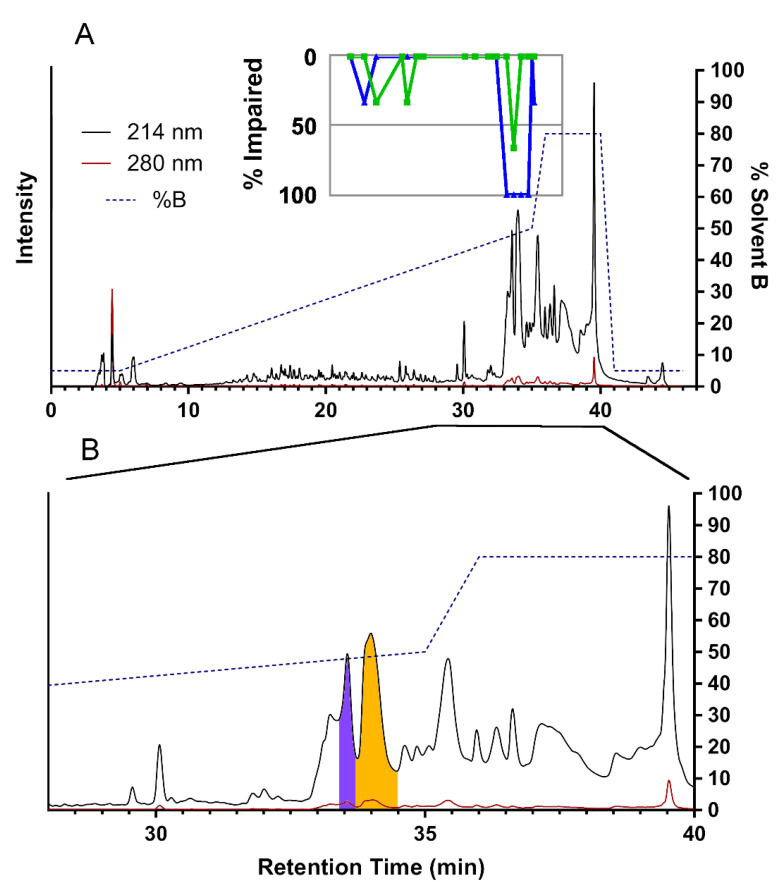
Reversed-phase high-performance liquid chromatography (RP-HPLC) fractionation and assay of insecticidal activities of *H. rufovarius* venom fractions. (**A**) Entire HPLC chromatogram, showing the vast majority of venom components eluting between 40–80% solvent B. Results of insecticidal assays are shown in the inset (green = paralysis at 5 min, blue = paralysis at 30 min); (**B**) Enlargement of the region from 28 to 40 min. Fraction f92 is highlighted in purple and f93 in orange.

To identify toxins responsible for insect paralysis, we injected sheep blowflies (*Lucilia cuprina*) with either water (negative control) or RP-HPLC fractions after drying and resuspension in water. For this purpose, 19 RP-HPLC fractions were selected that presented well-resolved peaks in the RP-HPLC chromatogram and yielded interpretable MALDI-TOF mass spectra. Of the tested fractions, 15 produced little or no effect, but four consecutive fractions (f91–94) displayed paralytic and lethal effects (Figure 3). At 30 min post-injection, all three f91-injected flies were completely paralysed, and each of the f92–94-injected groups displayed two completely paralysed flies and one with paralysis of the four hind legs.

Based on absorbance at 280 nm, the f92 and f93 venom fractions administered to flies were estimated to have a protein concentration of 0.476 and 0.993 mg/mL, respectively. Since 2 μL of each venom fraction was injected into flies, this suggested that 0.95 μg and 1.98 μg of f92 and f93, respectively, were sufficient to cause the observed insecticidal effects.

### 2.3. Toxin Mass Determination Using Mass Spectrometry

**Figure 4:**
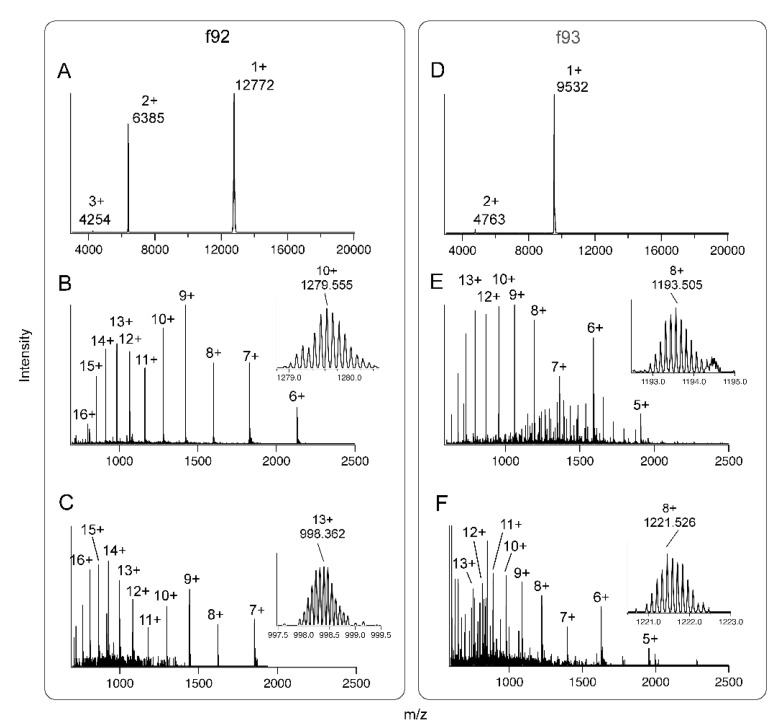
Mass spectra of insecticidal fractions f92 and f93. (**A**) Linear mode MALDI-TOF-MS spectrum of f92; (**B**) liquid chromatography–tandem mass spectrometry (LC-MS/MS) spectrum of native f92; (**C**) LC-MS/MS spectrum of f92 after reduction and alkylation with 2-iodoethanol; (**D**) Linear mode MALDI-TOF-MS spectrum of f93; (**E**) LC-MS/MS spectrum of native f93; (**F**) LC-MS/MS spectrum of f93 after reduction and alkylation with 2-iodoethanol. Insets in (**B**–**F**) show enlargements of one charge state with isotopic distributions visible and peak masses labelled.

We hypothesised that the consecutively eluting fractions f91–94 might contain a single toxin or variants of the same toxin. However, MS analysis revealed distinct but different masses for f92 and f93 (Figure 4), whereas the MS spectra of f91 and f94 were more complex and indicative of multiple components.

Linear mode MALDI-TOF-MS indicated that f92 was a toxin with an average mass close to 12,770 Da (Figure 4A), whereas f93 had an average mass close to 9,530 Da (Figure 4D). Reflectron-positive mode MALDI-TOF-MS did not yield interpretable spectra for any of f91–f94, suggesting that none of these fractions contained peptides in the mass range 1–4 kDa. Since no signal was obtained for f91 or f94 using either linear or reflectron mode MS, we focussed subsequent work on f92 and f93.

We investigated the mass and cysteine content of toxins in f92 and f93 in more detail using a triple-TOF liquid chromatography–tandem mass spectrometry (LC-MS/MS) instrument with higher mass accuracy than linear mode MALDI-TOF. For f92, the MS spectrum contained well-resolved charge states from *z* = 6 through *z* = 16 (Figure 4B). As expected for a toxin of this size on this instrument, the monoisotopic peaks were too low in signal to measure accurately, so we used the highest peak of the *z* = 10 ion to calculate a more exact mass of 12,786.3 Da for this toxin. Reduction and alkylation of cysteine residues with 2-iodoethanol shifted this peak mass to 12,966.1 Da (Figure 4C), which was consistent with the alkylation of four cysteines that formed two disulfide bonds. For f93, charge states from *z* = 5 through *z* = 15 were obtained, and the mass of the toxin was estimated to be 9540.5 Da (Figure 4E). Reduction and alkylation with 2-iodoethanol shifted the mass to 9763.7 Da (Figure 4F). The observed 223.2 Da difference suggested that this toxin contained five cysteines, four of which formed two disulfide bonds while one featured a free sulfhydryl group. No alternative explanation, including the formation of disulfide-linked homo- or heterodimers, was consistent with the MS data obtained. These data indicated the major components of f92 and f93 were polypeptides that contained five and four cysteine residues, respectively.

### 2.4. A CUB Domain Protein and a Cystatin Are the Primary Components of Insecticidal Venom Fractions

To identify the primary structure of the insecticidal toxins in f92 and f93, we combined venom-gland transcriptomics with tandem MS. Due to the high masses of the toxins, we did not attempt sequence identification from intact polypeptides, which was typically inefficient in our experimental setup for toxins >6 kDa, and instead relied on tandem MS data from reduced, alkylated, and trypsin-digested toxins. To create an amino acid sequence database, total RNA was extracted from the venom glands of one male and one female *H. rufovarius* and RNA-Seq was carried out on polyA+ RNA using the Illumina sequencing platform (Illumina, San Diego, CA, USA). This generated 10,109,098 (2 × 150 bp) reads, which were trimmed and assembled using Trinity 2.2.0 [16] and CLC Genomics Workbench (Qiagen, Hilden, Germany) to create a library of 473,072 contigs, from which a library of 1,127,926 translated open reading frames were extracted using TransDecoder [16] and used as a database to compare to MS/MS data. LC-MS/MS data obtained from f92 and f93 after reduction, alkylation, and trypsin digestion was then compared with the generated database using Paragon Algorithm in ProteinPilot software (SCIEX, Framingham, MA, USA).

**Figure 5:**
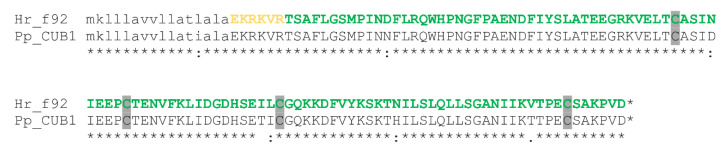
Amino acid sequence of the *H. rufovarius* CUB protein 1 (GenBank accession number MN686343) compared to *P. plagipennis* venom CUB domain protein 1. Tryptic peptides detected with >95% confidence are shown in green, and peptides detected with 50–95% confidence are shown in yellow. Since multiple overlapping tryptic peptides are detected that cover the entire putative mature sequence, the entire mature sequence is coloured. The precise tryptic peptides detected are listed in Supplementary Table S1. Predicted signal sequences are shown in lower case and the putative mature protein in upper case. Cys residues are highlighted in grey, the star at the end of each sequence represents a stop codon, while symbols beneath the alignment represent similarity in CLUSTALW format.

LC-MS/MS, due to its very high sensitivity, identified traces of 58 *H. rufovarius* proteins in f92 (Supplementary Table S1). Of these, TRINITY_DN14516_c4_g1_i3 appeared likely to be the most abundant component of the fraction, as evidenced by the very high number of distinct peptide ion variants detected (499), the fact that the seven tryptic peptides detected with highest intensity all arose from this sequence, and its close match to the features observed in MALDI-TOF spectra. This sequence, which is shown in Figure 5, features a translated open reading frame of 130 amino acids that begins with a 16-amino acid predicted secretion signal sequence (SignalP 4.1 D-score = 0.848), contains four cysteine residues, and is followed by a stop codon.

The LC-MS/MS identification was extremely confident (>99.99%), with 499 distinct peptide ion variants detected, representing 94.7% of the predicted mature sequence detected at >95% confidence. The predicted N-terminal mature sequence EKRKVR was detected at lower confidence (>50%), likely due to the numerous closely-spaced trypsin cleavage sites (i.e., C-terminal to Lys and Arg residues) in this region. The theoretical isotopic distribution of the amino acid sequence obtained with *z* = 10 had a peak mass of 1279.555, closely matching the observed value of 1279.633 Da (Figure 4C). This suggested that the amino acid sequence obtained exactly matched the isolated toxin, and that no post-translational modifications (PTMs) other than disulfide bond formation were present. The mature toxin sequence was used as a query to search GenBank’s non-redundant (nr) protein sequence database using the BLASTp algorithm. This search revealed that the identified toxin sequence shared 94% identity with venom CUB domain protein 1 from the assassin bug *P. plagipennis* [2], differing at only seven sites in the mature sequence (Figure 5).

**Figure 6:**
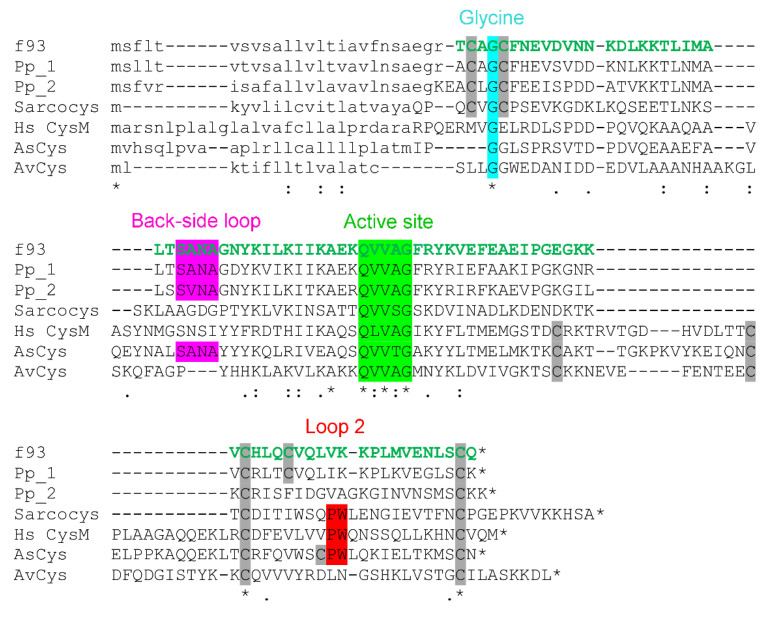
Alignment of *H. rufovarius* venom cystatin 1 (GenBank accession number MN686344) with known cystatins. Tryptic peptides detected with >95% confidence are shown in green. Since numerous tryptic peptides covering the entire mature sequence are detected, the entire sequence appears green. The precise tryptic peptides detected are listed in Supplementary Table S1. Primary structural characteristics are as indicated in Figure 5. It is aligned against Pp_1 and Pp_2, assassin bug *P. plagipennis* venom cystatin 1 and 2; Sarcocys, flesh fly sarcocystatin A (UniProt P1727.1); Hs CysM, human cystatin M (GenBank NP_001314.1), AsCys, copperhead snake *Austrelaps superbus* venom cystatin (GenBank FJ411278); and AvCys, orb spider *Araneus ventricosus* cystatin (GenBank AEV53625).

For f93, Paragon searches identified 70 amino acid sequences present (Supplementary Table S1), most of which likely represented fragments of serine proteases that may arise from venom self-digestion. However, the major component detected using MALDI-TOF corresponded to TRINITY_DN14757_c1_g1_i2, a sequence that was 111 amino acids long, beginning with a 25-residue signal sequence (SignalP 4.1 D-score = 0.79) and followed by a stop codon. The LC-MS/MS identification was extremely confident, with 205 distinct peptide ion variants detected with >95% confidence. These peptides covered 100% of the predicted mature sequence but 0% of the predicted signal peptide (Figure 6).

Consistent with LC-MS experiments before and after alkylation (Section 2.3), the mature sequence identified contained five cysteine residues. Again, the obtained sequence was likely to be identical to that of the isolated toxin, without PTMs other than disulfide bonds. The predicted peak mass of the isotopic distribution of the *z* = 8 ion for this sequence was 1193.505 Da, closely matching the observed peak mass of 1193.567 Da (Figure 4E). A BLASTp search of the identified sequence against GenBank’s nr database revealed it to have 75.6% identity with venom cystatin-like protein 1 from the assassin bug *P. plagipennis* [2] (Figure 6), and hence it was named *H. rufovarius* venom cystatin 1.

Although we attempted to further subfractionate the remainder of f92 and f93 using a diphenyl RP-HPLC column in order to purify each toxin to homogeneity, these experiments were not successful and resulted only in fractions of much lower (<0.045 mg/mL) concentration that did not display paralytic activity when injected into blowflies, probably due to the small amount of starting protein. However, the closely matching LC-MS/MS, MALDI-TOF-MS, and RNA-Seq data collected for both f92 and f93 suggested that one or both of the identified CUB domain protein and cystatin-like protein were major paralytic and lethal toxins that contributed to the insecticidal activity of *H. rufovarius* venom.

## 3. Discussion

In this study, we investigated the toxins involved in the paralytic action of assassin bug venom. Venom of the red tiger assassin bug (*H. rufovarius*) was fractionated by RP-HPLC, and four fractions were found to have paralytic effects when injected into blowflies. The main component present in one of these, f92, was found to share 94% identity with venom CUB domain protein 1 from the assassin bug *P. plagipennis*. The other, f93, shared 74% identity with venom cystatin-like protein 1 from the same species. This study is the first to examine the venom of *H. rufovarius*, and the first to attempt assay-guided fractionation combined with proteotranscriptomics to identify bioactive components of assassin bug venom.

The CUB domain is present in numerous extracellular and plasma membrane-associated multidomain proteins, many of which are developmentally regulated [17]. They are often protein recognition/binding and adaptor domains in these multidomain proteins. However, in the *H. rufovarius* protein, the CUB domain is unusually present in a “solitary” form as a 12.8 kDa protein, as is the case with the closely related orthologue in the venom of *P. plagipennis* (Figure 5). In *P. plagipennis* venom, CUB domain protein 1 is one of five solitary CUB domain proteins, and among the most abundant protein in the venom [10]. Contigs encoding similar solitary CUB domain proteins have also been reported in a cDNA library enriched for proteins selectively expressed in the venom gland of a related cimicomorphan heteropteran, the minute pirate bug *Orius laevigatus* [13], suggesting they may be present in a broad phylogenetic range of heteropteran venoms. While the phylogeny of Australian assassin bugs is poorly characterised, *P. plagipennis* and *H. rufovarius* both belong to the tribe Harpactorini in the subfamily Harpactorinae. Nevertheless, they belong to different genera and occupy different habitats (open foliage in the case of *P. plagipennis* compared with under tree bark for *H. rufovarius*). We found that a major venom protein of both species differed by only seven amino acid residues, most of which represented conservative replacements. This is consistent with strong purifying selection, for example, if a protein is important for paralysing prey. The lethal and paralytic effect of the *H. rufovarius* f92 provides the first evidence suggesting a toxic function of isolated CUB domain proteins.

The major component of the other insecticidal fraction isolated, *H. rufovarius* venom cystatin 1, is a member of the cystatin cysteine protease inhibitor family that occurs in diverse animals, plants, and micro-organisms [18]. Cystatins have been identified in the venoms of various snakes, including the Australian lowland copperhead (*Austrelaps superbus*) [19]. They also occur in spider venoms such as that of the orb-weaver spider (*Araneus ventricosus*) [20], as well as stingray venom [20], and tick saliva [19]. Cystatins make up as much as 9.8% of venom from the Gaboon viper *Bitis gabonica gabonica*, but the function of snake and spider venom cystatins is unknown [21]. It has been suggested that they may protect other venom components from prey proteases [21] or contribute to oral health like mammalian salivary cystatins [19]. However, *H. rufovarius* venom cystatin 1 was found to be the major proteinaceous component of a fraction that paralysed and killed flies (f93). This result suggests that cystatins may be paralytic or lethal toxin components themselves, at least in assassin bug venoms.

Structurally, assassin bug venom cystatins are similar to a class of short cystatins identified from insects, of which sarcocystatin-A from the flesh fly *Sarcophaga peregrina* is the archetype [22]. These similarities include a CXGC motif near the N-terminus and lack of the loop 1 region of short cystatins such as human cystatin M or venom cystatins AsCys and AvCys from the lowland copperhead snake *A. superbus* or the orb spider *A. ventricosus* (Figure 6). Interestingly, the predicted mature sequence of *P. plagipennis* venom cystatin 1 features five cysteine residues at the same position as the *H. rufovarius* protein, whereas *P. plagipennis* venom cystatin 2 and sarcocystatin-A are missing the fourth Cys residue of these proteins. Several structural features have been identified as important for the cysteine protease inhibition function of cystatins, such as a Gly residue near the N-terminus (Figure 6, blue) that allows the N-terminal motif to form a tight turn and fit in the substrate binding cleft of cysteine proteases. This Gly residue, and the QXVXG motif that is necessary for inhibitory activity [19], are conserved in both groups of cystatins. Other elements, such as the PW motif (loop 2), reported to be important for the integrity of the enzyme-binding surface [20], occur in sarcocystatin but not the assassin bug venom cystatins. Conversely, an Asn residue in the back-side loop (Figure 6, purple) that is reported to be necessary for inhibition of legumain by multiple human cystatins [23] is present in the assassin bug proteins but not sarcocystatin.

A major limitation of this study was that, although our data strongly suggested one or both of the identified proteins was a potent insecticidal toxin, it was impossible to unequivocally attribute the toxic effects of f92 and f93 to the major components we detected within them. In these adjacent fractions, it is possible that just one of these proteins possesses high potency and underlie the paralytic effects of all of f91–f93 even at low concentrations, beyond the limits of detection of the methods employed here. Alternatively, the observed insecticidal activity could be due to small molecules (<1 kDa) or high-molecular-mass toxins (>15 kDa) that were not well-resolved by our methods. Further studies are required to clarify the activity of the identified proteins, for example, by testing the activity of heterologously expressed versions of these proteins. Nevertheless, this study adds to our knowledge of the composition and activity of reduviid venoms and provides leading candidates for further investigation that may account for the insecticidal activity of assassin bug venom.

## 4. Materials and Methods

### 4.1. Sample Collection

*H. rufovarius* were collected from gum trees on private land in Brisbane, Australia. They were housed in individual containers to prevent cannibalism and fed crickets. Venom was collected from eight individuals of both sexes using the protocol of Walker et al. [4,10], through both electrostimulation and harassment. Briefly, bugs were restrained on a small platform with a rubber band and the proboscis was inserted into a P200 pipette tip with tweezers. For harassment, the bug was touched and gently grabbed with tweezers until venom was ejected. For electrostimulation, a mild stimulus train (5 ms pulses at 5 Hz of 15–25 V) was applied across the thorax using electrified tweezers dipped in saline and linked to a S40 electrostimulator (Grass Technologies, Middleton, WI, USA). In both cases, this involved recovering venom from a P200 pipette tip with water. The venom was stored at −20 °C until further use.

### 4.2. Sample Fractionation

Crude venom (0.5 mg as estimated using a NanoDrop™ spectrophotometer (Thermo Fisher, Waltham, MA, USA) obtained by electrostimulation was fractionated using a Nexera HPLC system (Shimadzu, Kyoto, Japan) running an Aeris PEPTIDE XB-C18 250 × 4.6 mm column (Phenomenex, Torrance, CA, USA). Venom components were eluted isocratically for 5 min using 5% solvent B (0.043% trifluoroacetic acid (TFA) in 90% acetonitrile (ACN)) in solvent A (0.043% TFA in water), followed by a gradient of 5 to 50% solvent B over 30 min, then 80% solvent B for 5 min, all at a flow rate of 0.75 mL/min. Absorbance was monitored at 214 and 280 nm.

### 4.3. Insecticidal Assays

Sheep blowflies (*Lucilia cuprina*) were used for insecticidal assays a day after hatching. Next, 2 μL of each venom fraction resuspended in 30 μL distilled water was injected into each fly using a 1 mL syringe in a hand microapplicator (Burkard Scientific, Rickmansworth, UK). Distilled water was used as a control (*n* = 5), and venom fractions were injected separately (*n* = 3), washing the syringe between fractions. Flies were scored as either alive, dead, or impaired (i.e., limited movement), by dropping the containers onto the bench (<1 cm drop) to encourage movement. Insecticidal fraction concentrations were determined using a NanoDrop™ 2000 spectrophotometer (Thermo Fisher, Waltham, MA, USA) on the Protein A_280_ setting.

### 4.4. Mass Spectrometry

MALDI-TOF-MS was used to determine the masses of molecules in the crude venom and venom fractions. Equal proportions (0.4 μL) of samples and α-cyano-4-hydroxycinnamic acid matrix (5 mg/mL in 70% ACN, 0.1% formic acid) were spotted on the plate, allowed to dry, then analysed on an AB SCIEX TOF/TOF 5800 with both reflectron positive and linear methods. LC-MS/MS was undertaken for the insecticidal HPLC fractions. For each sample, ~2–10 μg of sample was used, either untreated, reduced and alkylated, or reduced, alkylated, and trypsinised. Reduction and alkylation was achieved by incubating samples in 50 µL reduction/alkylation cocktail (48.75% ACN, 100 mM (NH_4_)_2_CO_3_ pH 11.0, 1% 2-iodoethanol, 0.25% triethylphosphine) at 37 °C for 1 h. After drying by vacuum centrifugation, trypsin digestion was performed on previously reduced and alkylated samples by incubation with 25 µL trypsin reagent (40 ng/µL sequencing-grade trypsin in 100 mM NH_4_HCO_3_ pH 8.0, 10% ACN) at 37 °C for 2 h and the reaction was quenched by the addition of 5% formic acid/50% ACN. After drying by vacuum centrifugation, samples were resuspended in 1% formic acid and analysed by LC-MS/MS using an Ekspert nanoLC 400 with a ChromXP C_18_-CL column (Eksigent; 150 mm × 0.3 mm, 3 μm) attached to an TripleTOF 6600 mass spectrometer (SCIEX, Waltham, MA, USA). A linear gradient of 1–30% solvent B in solvent A over 35 min (flow rate 5 μL/min) was used to elute peptide fragments. MS^1^ scans were collected between 350 and 1800 *m*/*z*, and precursor ions in the *m*/*z* 80–1400 range. Precursor peptides with charge states between +2 and +5, and >100 counts/s, were selected for fragmentation using the dynamic “rolling collision energy” option.

### 4.5. Transcript/Sequence Generation

A venom-gland transcriptome was generated from two adult *H. rufovarius* (one male and one female). The entire venom-gland complex (including anterior and posterior main glands and accessory gland) was removed by dissection and stored in RNAlater (Ambion). Total RNA was extracted using a DNeasy kit (Qiagen, Hilden, Germany), and mRNA was isolated using a Dynabeads mRNA DIRECT Kit (Thermo Fisher, Waltham, MA, USA). The mRNA was sequenced with an NextSeq instrument (Illumina, San Diego, CA, USA) at the Institute for Molecular Bioscience Sequencing Facility. Transcripts were then assembled in Trinity 2.2.0 [16] using the default trimming parameters. Raw transcriptome data was submitted to the National Centre for Biotechnology Information (NCBI) Sequence Read Archive database, where it was allocated as BioProject PRJNA685014 and BioSample SAMN17074910. Open reading frames >30 amino acids were extracted and translated from each contig using TransDecoder [16]. Redundancy was removed from the generated amino acid sequences using CD-HIT with a similarity threshold of 100%. Common MS contaminants were added to the file, which was then used as a database for searches employing the Paragon [24] and ProtGroup algorithms in ProteinPilot software (SCIEX, Waltham, MA, USA). BLASTp searches were run with the mature peptide sequences to identify homologues. Signal peptides were predicted using SignalP 4.1 [25].

## Supplementary Materials

The following are available online at https://www.mdpi.com/2072-6651/13/1/3/s1, Table S1. Results of LC-MS/MS experiments. Sheet 1: Summary of protein sequences detected in f92. Sheet 2: Summary of peptides detected in f92. Sheet 3: Summary of protein sequences detected in f93. Sheet 4: Summary of peptides detected in f93.
